# Laparoscopic surgery for double gastrointestinal stromal tumor of the stomach: a report of two cases

**DOI:** 10.1186/1477-7819-12-76

**Published:** 2014-03-29

**Authors:** Nicola de’Angelis, Riccardo Memeo, Valeria Zuddas, Dalila Mehdaoui, Daniel Azoulay, Francesco Brunetti

**Affiliations:** 1 Digestive and Liver Transplant Surgery Unit, Henri-Mondor Hospital, Université Paris Est, 51 avenue du Maréchal de Lattre de Tassigny, 94010 Créteil, France; 2 Unit of Histopathology, Henri-Mondor Hospital, Université Paris Est, Créteil UPEC 94010, France

**Keywords:** Gastrointestinal stromal tumors, Laparoscopic surgery, Gastrectomy

## Abstract

Gastrointestinal stromal tumors (GISTs) are mesenchymal tumors that originate from interstitial cells of Cajal or their stem cell-like precursors. Generally, GISTs have specific c-KIT gene mutations. The incidence of GISTs is estimated to be 10 to 20 cases/one million individuals, and GISTs typically affect people over 50 years of age. The majority of GISTs are solitary. However, multifocal GISTs have been observed, especially in children. We report on two unusual adult cases of double GISTs that were treated by laparoscopic surgery. The first patient presented a polypoid mass of the fundus and a second isolated smaller tumor in the posterior wall of the lesser curvature of the stomach. A histopathological examination confirmed that both tumors were GISTs and were *c-KIT-*positive. A total laparoscopic gastrectomy was performed. In the second patient, GISTs were identified at the level of the fundus and the greater curvature of the stomach. A laparoscopic partial sleeve gastrectomy was performed. Both surgeries were successful with no complications or relapses at three to five years following surgery.

## Background

Gastrointestinal stromal tumors (GISTs) are mesenchymal neoplasms of the gastrointestinal tract that account for approximately 0.1% to 3% of all gastrointestinal malignancies [1]. GISTs occur more frequently in the stomach (60% to 70%), followed by the small intestine (20% to 30%), the colon and the rectum (5%), and the esophagus (less than 5%) [1,2].

In most cases, GISTs originate from interstitial cells of Cajal, a complex cellular network that regulates gastrointestinal motility, or mesenchymal stem cells of the mesentery or omentum, which transform into the malignant phenotype that expresses the proto-oncogene *c-Kit* (*CD 117*) and less frequently the *platelet-derived growth factor receptor-α* (*PDGFRA)*[3,4].

Small GISTs (less than 2 cm in diameter) are usually asymptomatic and are detected during investigations performed for other unrelated diseases. Conversely, GISTs that are greater than 2 cm are generally associated with clinical signs and symptoms, such as nausea, vomiting, abdominal pain, obstruction, abdominal mass, anemia and melena [2,3]. Diagnosis and staging are usually based on abdominal ultrasonography, computed tomography (CT) scanning, and/or magnetic resonance imaging (MRI).

Clinically, GISTs vary from virtually benign diseases to aggressive tumors (20% to 30% of cases) with a five-year survival rate that ranges from 35% to 65% depending on the tumor size, the mitotic index (the mitotic count/50 high-power fields, HPFs) and the tumor location [5]. Moreover, 50% of GISTs may be metastatic at presentation, and the most common sites of metastases are the peritoneum and the liver [1,3,6]. Over 95% of primary GISTs are solitary tumors; however, case reports and small case series have reported multiple primary lesions, which were restricted to the familial or pediatric forms, and district syndromes (type 1 neurofibromatosis, von Recklinghausen disease and Carney’s syndrome) [1,3,7].

Complete surgical resection is the only curative treatment for GISTs [8]. However, the recent introduction of specific pathogenesis-targeted treatments with a *Kit* tyrosine kinase inhibitor, imatinib mesylate (Glivec®, Novartis Pharma S.A.S, France), has resulted in significant improvements in non-resectable patients (a palliative cure), and it has been successfully used as adjuvant therapy for tumors with a high risk of relapse [4].

## Case Presentation

We report two unusual cases of double GISTs of the stomach in two female adult patients.

### Case report 1

**Figure 1:**
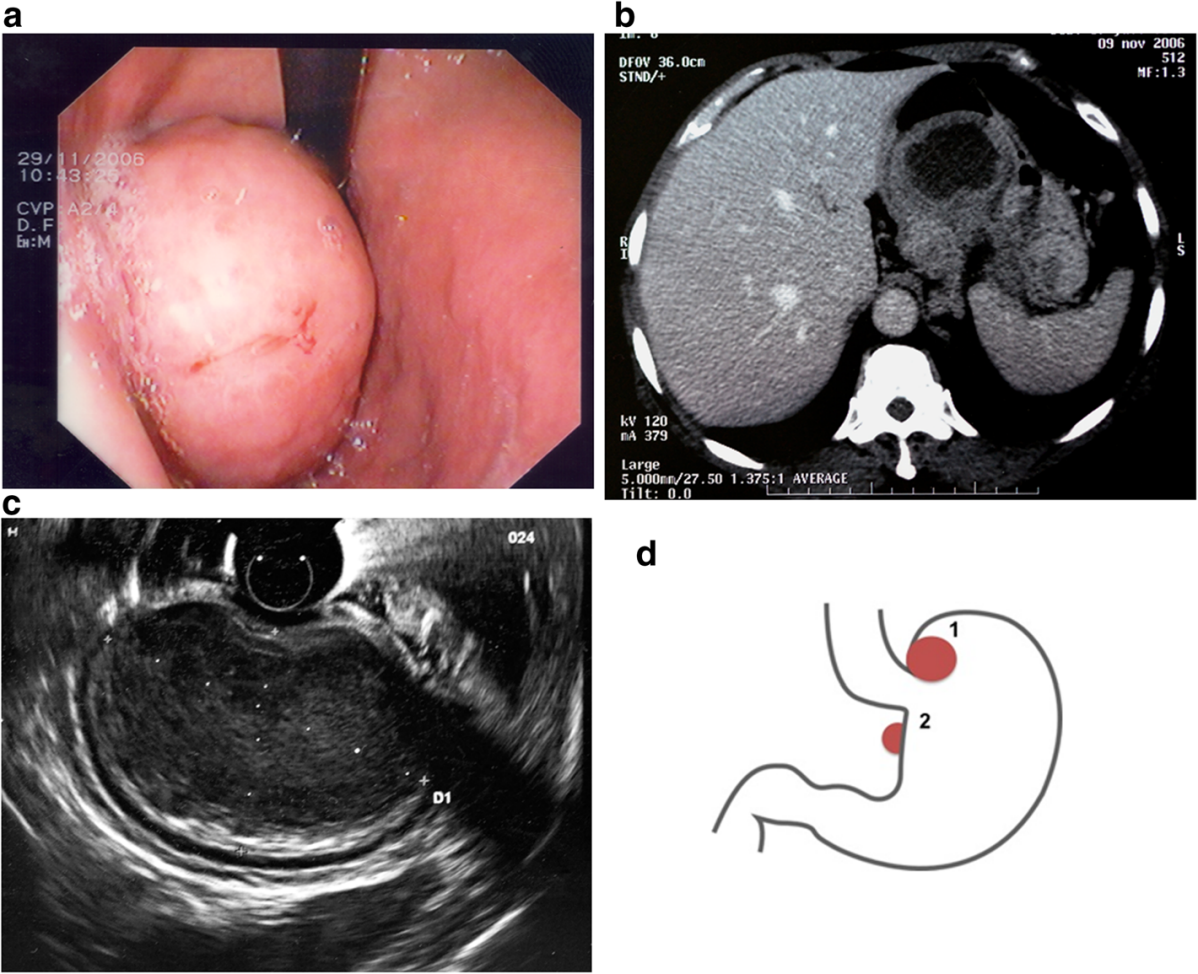
**Imaging and schematic representation of double GIST in case report 1. a**. Case report 1. Esophagogastroscopy: a polypoid lesion of the fundus, which was suggestive of a GIST. **b**. Case report 1. CT scan imaging: a tumoral mass under the cardia region was 8 × 7 × 6 cm without sites of distant metastasis. **c**. Case report 1. Echoendoscopy with fine needle aspiration (EUS-FNA) imaging*.* Visualization of the gastric tumor. **d**. Case report 1. A schematic representation of the locations of the double GISTs. Tumor 1 was 7 × 6 × 6 cm and was observed to be a polypoid lesion on the fundus of the stomach. Tumor 2 was discovered intra-operatively, was 2.5 × 1 × 1 cm and was located in the posterior wall of the lesser curvature.

A 59-year-old female patient with a history of hypertension, breast cancer and type II diabetes visited her general practitioner because of a stomachache, dysphagia to solids, weight loss (approximately 13 kg), and general fatigue that began three months earlier. She was referred to a gastroenterologist for further examinations. An esophagogastroscopy was performed and indicated chronic gastritis of the antrum and a polypoid lesion of the fundus, which was suggestive of a GIST (Figure 1a). A CT scan was performed for diagnosis and staging. The CT showed a tumoral mass under the cardia region that was 8 × 7 × 6 cm without sites of distant metastasis (Figure 1b). Echoendoscopy with fine needle aspiration (EUS-FNA) confirmed the presence of the mass (Figure 1c), but an immunohistochemical examination was not specific for a GIST. The serological tumoral biomarkers (CA 15.3, CA 125, CEA and CA 19.9) were negative.

The patient planned to undergo laparoscopic surgery for the resection of the tumor by sleeve gastrectomy. However, the intra-operative laparoscopic ultrasound uncovered another lesion that was isolated from the mass of the fundus and located in the posterior wall of the lesser curvature of the stomach (Figure 1d). Therefore, for anatomical reasons, the planned surgical technique was changed, and a total laparoscopic gastrectomy with Roux-en-Y reconstruction was performed for the resection of both tumors.

The histopathological and immunohistochemical examinations revealed two isolated GISTs (7 × 6 × 6 cm and 2.5 × 1 × 1 cm) that were *c-kit-* and *CD34-*positive and *SMMA*- and *PDGFRA*-negative. A mutation in the 11th exon of the *KIT* gene was observed in only one lesion. In the first tumor, two to four mitoses per 50 HPF were counted, and five mitoses per 50 HPF were found in the second tumor. The resection margins (R0) and the lymph nodes were free of neoplasia (0/34). The postoperative course was uneventful, and the patient was discharged on the 14th postoperative day. Adjuvant therapy was proposed with Glivec® 400 mg/day for 12 months. After five years of follow-up, the patient is clinically and radiographically disease-free.

### Case report 2

**Figure 2:**
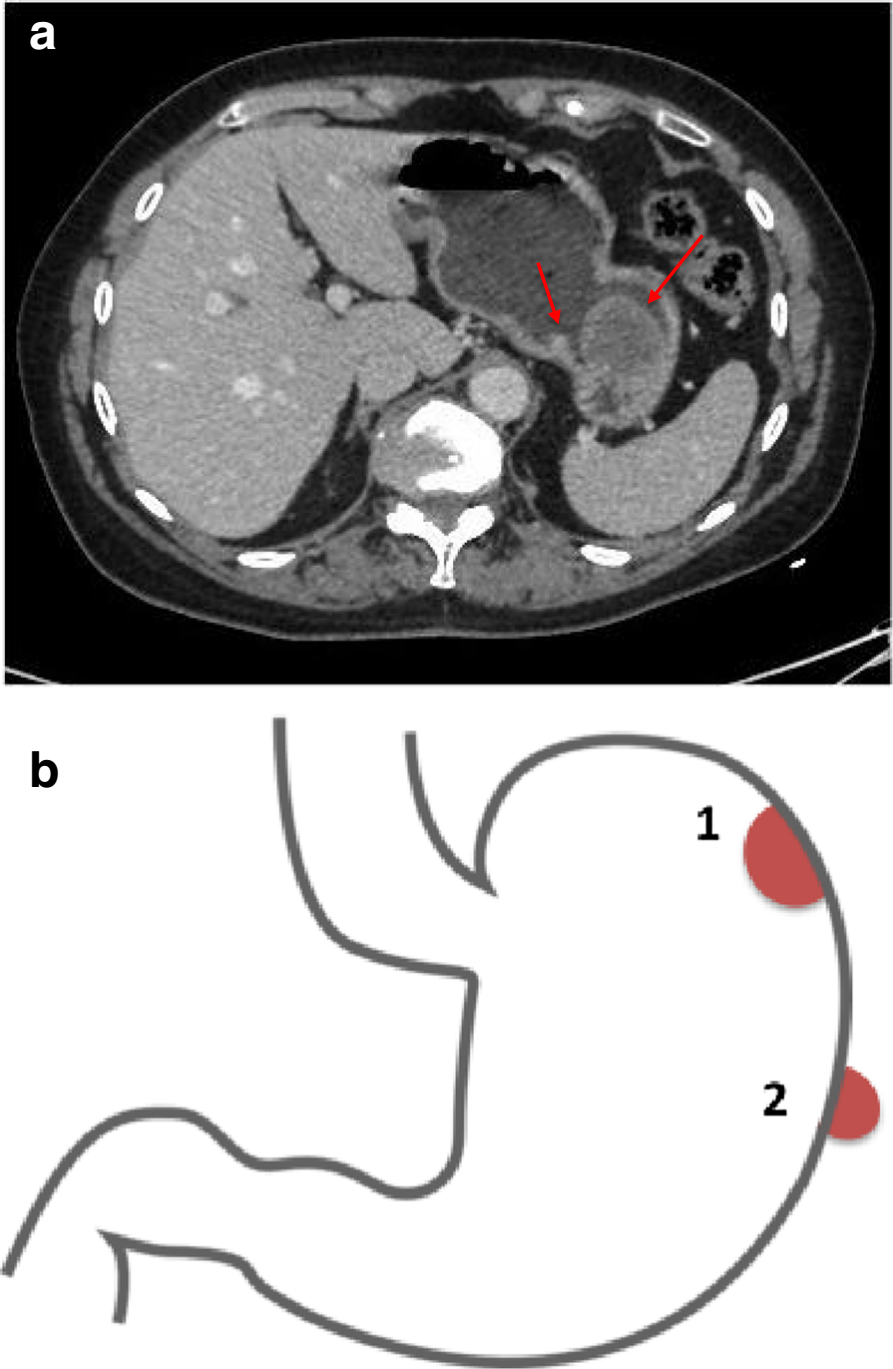
**Imaging and schematic representation of double GIST in case report 2. a**. Case Report 2. CT scan imaging: the tumoral lesions (the red arrows) were 6 × 5 × 2.5 cm and 2.5 × 1.5 × 1 cm. **b**. Case report 2. A schematic representation of the location of the double GISTs. Tumor 1 was an intramural lesion at the level of the fundus of the stomach (6 × 5 × 2.5 cm), whereas tumor 2 was an extraluminal gastric mass located in the middle of the greater curvature (2.5 × 1.5 × 1 cm).

A 62-year-old female patient visited her general practitioner because of a stomachache, chronic diarrhea, occasional melena and weight loss (approximately 7 kg). An esophagogastroscopy was performed and revealed a polypoid lesion of the fundus, which was suggestive of a GIST. A CT scan was performed for diagnosis and staging. The CT confirmed the intramural lesion at the level of the fundus of the stomach (6 × 5 × 2.5 cm) and showed another extraluminal gastric mass that was located in the middle of the greater curvature (2.5 × 1.5 × 1 cm) (Figure 2a,b). No sites of distant metastasis were observed. EUS-FNA was performed on both tumors. The immunohistochemical examinations were specific for double GISTs.

The patient underwent laparoscopic resection by partial sleeve gastrectomy.

The histopathological and immunohistochemical examinations revealed two isolated GISTs (5.5 × 5 × 2.5 cm and 2 × 1 × 1 cm) that were *c-kit-* and *CD34-*positive and *SMMA*-negative. Mutations in the 9th and 11th exons of the *KIT* gene were observed in the first tumor, whereas a mutation in the 9th exon of the *KIT* gene was present in the second tumor. In the first tumor, three mitoses per 50 HPF were counted, and two mitoses per 50 HPF were found in the second tumor. The resection margins (R0) were free of neoplasia. The postoperative course was uneventful, and the patient was discharged on the sixth postoperative day. Adjuvant therapy was proposed with Glivec® 400 mg/day for 12 months. After three years of follow-up, the patient is clinically and radiographically disease-free.

## Discussion

GISTs are rare neoplasms of the gastrointestinal tract, which occur mostly as solitary tumors [1,6]. We report two unusual cases of double synchronous lesions that we observed among our series of 48 consecutive gastric resections for GISTs from 2005 to 2013. To our knowledge, this is the only case report in the literature of double gastric GISTs in adult patients after the one published by Golabek-Dropiewska and coworkers in 2009 [7].

Both our case patients had no history of familial GISTs, no diagnoses of GIST-related district syndromes, or any signs of metastatic spreading from a single primary GIST. Moreover, the patterns that were detected in the histopathological and immunohistochemical analyses supported the diagnosis of double sporadic GISTs. Particularly, the synchronous location in the proximal stomach, the close proximity and the demonstration of different *kit* mutations in the individual lesions suggested the presence of distinct subsets of gastric GISTs of an unknown etiology. As reported in the literature, the majority (approximately 79%) of cases of multifocal and closely associated GISTs that present *c-kit*^*+*^ and *CD34*^*+*^ phenotypes are initiated by different types of somatic *KIT* exon 9 and 11 mutations [9-11]. In the case of multiple mutations, they may be primary or, more frequently, acquired secondary mutations, which occur independently and randomly in individual GIST cells.

Surgical treatment is indicated in the case of a non-metastatic GIST >2 cm, and the standard primary treatment is complete resection with recommended 1-cm negative margins without tumor rupture. Microscopic negative margins are sufficient to ensure R0 resection [6,8,12]. The laparoscopic approach can be safely used for GIST resection; however, it is debatable whether its application is limited by the location and the size of the tumor [13].

Radiographic assessment is a prerequisite for correct surgical planning. In the initial investigation, esophagogastroscopy and trans-abdominal ultrasonography are undertaken. Then, in patients with gastric resectable GISTs, EUS-FNA and a CT scan with a “stomach protocol” [14] are performed to study the local anatomy and the histopathological analysis and to rule out the presence of metastatic spread. A CT scanner may have limitations in the assessment of small GISTs, whereas it has greater specificity and sensitivity for larger GISTs [15]. In case of an allergy to contrast media or for questions regarding liver metastases, MRI is useful to determine the relationship between the tumor and the adjacent organs [16].

In Case Report 1, we performed different diagnostic imaging techniques; however, only the larger GIST was correctly detected. Most likely due to its small size, the second GIST was not identified even by EUS-FNA. During surgery, we performed intra-operative laparoscopic ultrasound to evaluate the relationship between the tumor and the cardias. This procedure indicated the presence of another small lesion in the posterior wall close to the cardias. The discovery of a second tumor and the proximal location (<1 cm) from the gastro-esophageal (GE) junction required a total laparoscopic gastrectomy to guarantee an en-block excision with R0 resection margins and prevent stenosis of the GE junction. Moreover, we performed an extensive lymphadenectomy, even though this procedure is not recommended, because we did not know peri-operatively the nature of the second tumor. In this patient, the anatomical characteristics of the double GISTs determined the surgical choice, which led to a more invasive surgery that was counterbalanced by the achievement of the oncological outcomes.

In Case Report 2, the correct detection of both GISTs pre-operatively and their location at the greater curvature allowed us to perform a laparoscopic sleeve gastrectomy, which is less invasive and associated with a shorter recovery.

In both patients, the laparoscopic approach was selected regardless of the tumor size and the tumor location. The patient positioning and the location of the trocars were similar to those used in laparoscopic surgeries for early gastric cancers and Toupet fundoplication. The location of the trocars was adjusted to the left and proximally for lesions of the fundus of the stomach and distally when lesions were located toward the antrum. Before initiating the resection, we systematically performed an intra-operative laparoscopic ultrasound to better locate small lesions. If needed, intra-operative endoscopy can be used to support the surgical excision.

## Conclusion

In conclusion, this case report presents the surgical treatment of two unusual double gastric GISTs in adult patients. The laparoscopic approach was confirmed as a safe and effective technique, regardless of the size and the location of the tumor, when performed by skilled surgeons who work in a high-volume center.

## Consent

Written informed consent was obtained from the patient for publication of this case report and any accompanying images. A copy of the written consent is available for review by the Editor-in-Chief of this journal.

## Abbreviations

CT: Computed tomography scanning; EUS-FNA: Echoendoscopy with fine needle aspiration; GE: Gastro-esophageal; GIST: Gastrointestinal stromal tumors; MRI: Magnetic resonance imaging; PDGFRA: Platelet-derived growth factor receptor-α.

## Competing interest

The authors report no conflicts of interest. This study received no funding or financial support.

## Authors’ contributions

NdeA, FB and RM contributed to the study conception, the data analysis, the data interpretation, and the drafting and final revisions of the manuscript. DM and VZ contributed to the data collection, the data analysis and the drafting of the manuscript. DA contributed to the study conception and the final revision of the manuscript. All of the authors approved the final version of the manuscript.
